# A new system for parallel drug screening against multiple-resistant HIV mutants based on lentiviral self-inactivating (SIN) vectors and multi-colour analyses

**DOI:** 10.1186/1742-6405-10-1

**Published:** 2013-01-03

**Authors:** Maria M Prokofjeva, Kristoffer Riecken, Pavel V Spirin, Dimitriy V Yanvarév, Arne Düsedau, Bernhard Ellinger, Boris Fehse, Carol Stocking, Vladimir S Prassolov

**Affiliations:** 1 Engelhardt-Institute of Molecular Biology, Moscow, Russia; 2 Research Dept. Cell and Gene Therapy, Clinic for Stem Cell Transplantation, UCCH, University Medical Center Hamburg-Eppendorf (UKE), Hamburg, Germany; 3 Heinrich-Pette-Institute, Leibniz Institute for Experimental Virology, Hamburg, Germany; 4 European ScreeningPort, Hamburg, Germany; 5 Moscow Institute of Physics and Technology (MIPT), Moscow, Russia

**Keywords:** HIV, Drug resistance, Drug screening, Lentiviral vectors

## Abstract

**Background:**

Despite progress in the development of combined antiretroviral therapies (cART), HIV infection remains a significant challenge for human health. Current problems of cART include multi-drug-resistant virus variants, long-term toxicity and enormous treatment costs. Therefore, the identification of novel effective drugs is urgently needed.

**Methods:**

We developed a straightforward screening approach for simultaneously evaluating the sensitivity of multiple HIV gag-pol mutants to antiviral drugs in one assay. Our technique is based on multi-colour lentiviral self-inactivating (SIN) LeGO vector technology.

**Results:**

We demonstrated the successful use of this approach for screening compounds against up to four HIV gag-pol variants (wild-type and three mutants) simultaneously. Importantly, the technique was adapted to Biosafety Level 1 conditions by utilising ecotropic pseudotypes. This allowed upscaling to a large-scale screening protocol exploited by pharmaceutical companies in a successful proof-of-concept experiment.

**Conclusions:**

The technology developed here facilitates fast screening for anti-HIV activity of individual agents from large compound libraries. Although drugs targeting gag-pol variants were used here, our approach permits screening compounds that target several different, key cellular and viral functions of the HIV life-cycle. The modular principle of the method also allows the easy exchange of various mutations in HIV sequences. In conclusion, the methodology presented here provides a valuable new approach for the identification of novel anti-HIV drugs.

## Background

Introduction of combined antiretroviral therapy (cART) has dramatically altered the fate of HIV infected patients. In most cases, AIDS has changed from a fatal into a chronic, but manageable disease. Today, more than 25 drugs representing different classes of antiviral agents are available for combined therapy [1].

Despite this significant progress, there is still a strong and urgent need to develop novel anti-HIV agents. The main reasons for that are the following: (i) HIV has shown a very high capacity to acquire adaptive mutations. In fact, most HIV-infected individuals carry viruses that are resistant to at least one of the currently used drugs [2]. (ii) Since cART does not lead to HIV elimination, patients have to take drug combinations for their whole life. Therefore, in addition to short-term toxicity of any of the individual drugs and, potentially, their combination, many patients will face long-term adverse effects, such as liver and renal toxicity. This is associated with decreasing patient compliance, which in turn increases the risk of appearance of additional HIV mutants [2-4].

Thus, novel drugs should ideally combine lower toxicity with better anti-HIV efficacy, particularly against drug-resistant strains. To identify those drugs, easy and straightforward screening systems are highly desirable. Two drug-screening systems are frequently used. The first relies on direct drug-testing on purified HIV enzymes. That system, however, does not evaluate critical parameters, i.e. a drug’s ability to penetrate into cells, compound stability, or cytopathic drug effects. Moreover, only wild-type enzymes are usually tested. The second system utilises primary virus isolates and is therefore much more informative. However, its use for compound screening approaches is strongly limited by the rigid safety regulations necessary for using infectious HIV. As a consequence, only defined (wild-type) viruses can be used in most cases.

The use of recombinant HIV vector particles to test drug sensitivity of individual, patient-specific virus variants has been previously proposed [5,6]. We have extended this principle by combining it with our modular, multi-colour LeGO vector system [7]. Based thereon, we could simultaneously assess several HIV gag-pol mutants in single assays. In addition, by adapting the system to use ecotropic viral particles, the assay could be used in a Biosafety Level 1 environment [8]. We show here that the modular recombinant vector system facilitates parallel drug-testing and compound screening on up to four HIV variants at a time.

## Results and discussion

### *Simultaneous analysis of various* gag-pol *mutants in single assay*

**Figure 1:**
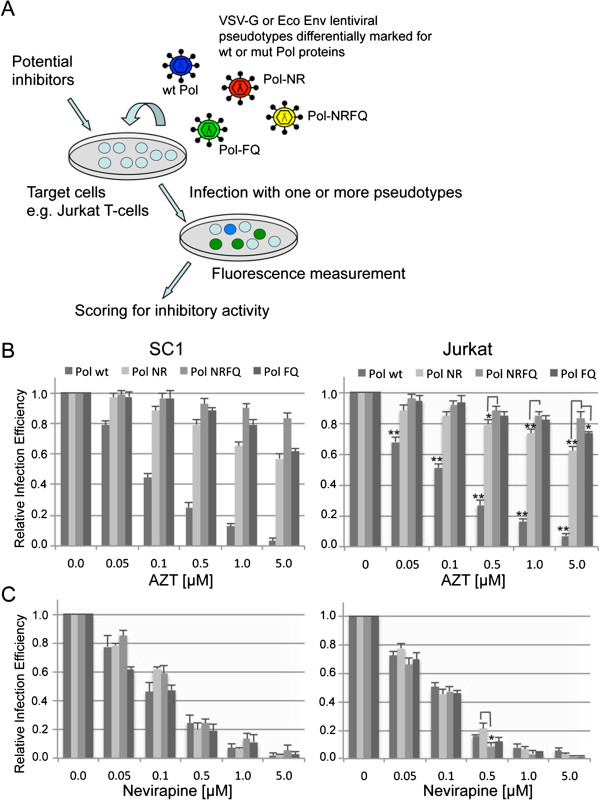
**Experimental outline and proof-of-principle.** (**A**) Based on the use of multi-colour LeGO vectors, drug-sensitivity of several (here: four) RT variants can be assessed simultaneously. (B + C) For proof of principle, two established antiviral drugs (B: AZT, C: Nevirapine) were tested in independent assays against four RT variants (wild-type, two double mutants [D67N/K70R = NR, T215F/K219Q = FQ] and one quadruple mutant [D67N/K70R/T215F/K219Q = NRFQ]) pseudotyped with VSV-G protein in two different cell lines (Left panels: SC1 cells, right panels: Jurkat cells). As expected, RT mutants, but not wild-type RT were highly resistant towards AZT (**B**). In contrast, all RT variants showed comparable sensitivity against Nevirapine (**C**). Data points represent mean values from three independent measurements. P-values that were significantly different between all other data groups, or only for those connected by lines, are denoted with *, for values < 0.05 or **, for values < 0.01.

Screening of potential novel drugs is time-consuming and expensive. Consequently, concurrent compound testing ideally targeting different molecules is highly desirable in order to save time and money. We designed an experimental setting that allows us to perform compound screening for various HIV wild-type and mutant genes in parallel (Figure 1A). For proof of principle we used three well-known *gag-pol* mutants [9].

The proposed system is based on the use of lentiviral LeGO vectors encoding fluorescent proteins with easily distinguishable emission spectres, with each given fluorescent colour corresponding to a defined virus variant, in our case different *gag-pol* variant (mutant or wild-type). LeGO vectors of different colours are then used to transduce target cells of choice in the presence of known HIV inhibitors or compounds to be tested. Consequently, sensitivity of a given HIV (*gag-pol*) variant to an applied drug will result in the loss of infectivity for the given construct and thus the absence of the corresponding colour in the transduced cell population. Thus, the efficiency of a given antiviral compound can be quantitatively assessed using flow cytometry and/or fluorescence microscopy (Figure 1A).

### Generation of recombinant lentiviral particles containing mutant reverse transcriptase molecules

For proof of principle we constructed three mutant *gag-pol* genes, in which the coding sequences for reverse transcriptase (RT) contained amino acid substitutions; namely two *gag-pol* genes each with two mutations (D67N/K70R and T215F/K219Q) and one *gagpol* gene containing all four mutations (D67N/K70R/T215F/K219Q). All enzymes encoded by those mutated genes were previously shown to confer resistance against the nucleoside inhibitor azidothymidine (AZT) [9]. Mutant genes were cloned in the same expression plasmid as the wild-type *gagpol* gene normally used for production of recombinant lentiviral particles [10].

Mutant or wild-type *gag-pol* genes were transfected into 293 T cells in conjunction with plasmids encoding vesicular stomatitis virus (VSV) G-protein (replacing the lentiviral Env protein), the lentiviral rev protein, and the plasmid containing the LeGO-G2 vector, mediating expression of enhanced green fluorescent protein (eGFP). Independent of the *gag-pol* construct used, we obtained good vector titres in the range of 5×10^6^ to 5×10^7^ infectious particles per ml as estimated on HEK-293 T cells by FACS analysis (not shown). Concordantly, reasonable titres (1×10^5^ to 5×10^5^) were also obtained for all mutant and wild-type gag-pol proteins after pseudotyping with ecotropic retroviral Env proteins (as assessed on SC1 embryonic murine fibroblasts). Although these viral titers are sufficient for the assay presented here, viral vector particles can easily be concentrated using standard methods, if other cell types or mutations that impact on functions are used. These results also underline the flexibility of the system for testing different mutations and using different cell systems.

### Transduction assays allow antiHIV-compound testing

Using the setting described above, we tested two well-established anti-HIV drugs, namely the nucleoside inhibitor azidothymidine (AZT) and the non-nucleoside inhibitor Nevirapine on two different cell lines (murine fibroblasts SC-1 and human T cells Jurkat). To do so, we independently transduced target cells with VSV-pseudotyped LeGO-G2 vectors generated using one of the four different *gag-pol* genes (three mutants, one wild-type) in the presence of increasing concentrations of the two drugs. In both cell lines tested, AZT most strongly inhibited transduction of particles containing the wild-type RT as compared to mutant RT, as expected (Figure 1B). In contrast, Nevirapine suppressed transduction by all lentiviral particles harbouring any of the four RT variants (Figure 1C). In summary, this data proves that the given system can be used for testing drug efficacy against different drug-resistant HIV mutants.

### Simultaneous testing of different HIV mutants in single assays

In the next step, we asked whether we could simultaneously test several mutants in single assays. At the same time, we investigated the potential to use ecotropic Env protein for pseudotyping. Viral vectors pseudotyped with the ecotropic Env protein are unable to transduce human cells and are therefore classified as GMO of Biosafety Level 1.

**Figure 2:**
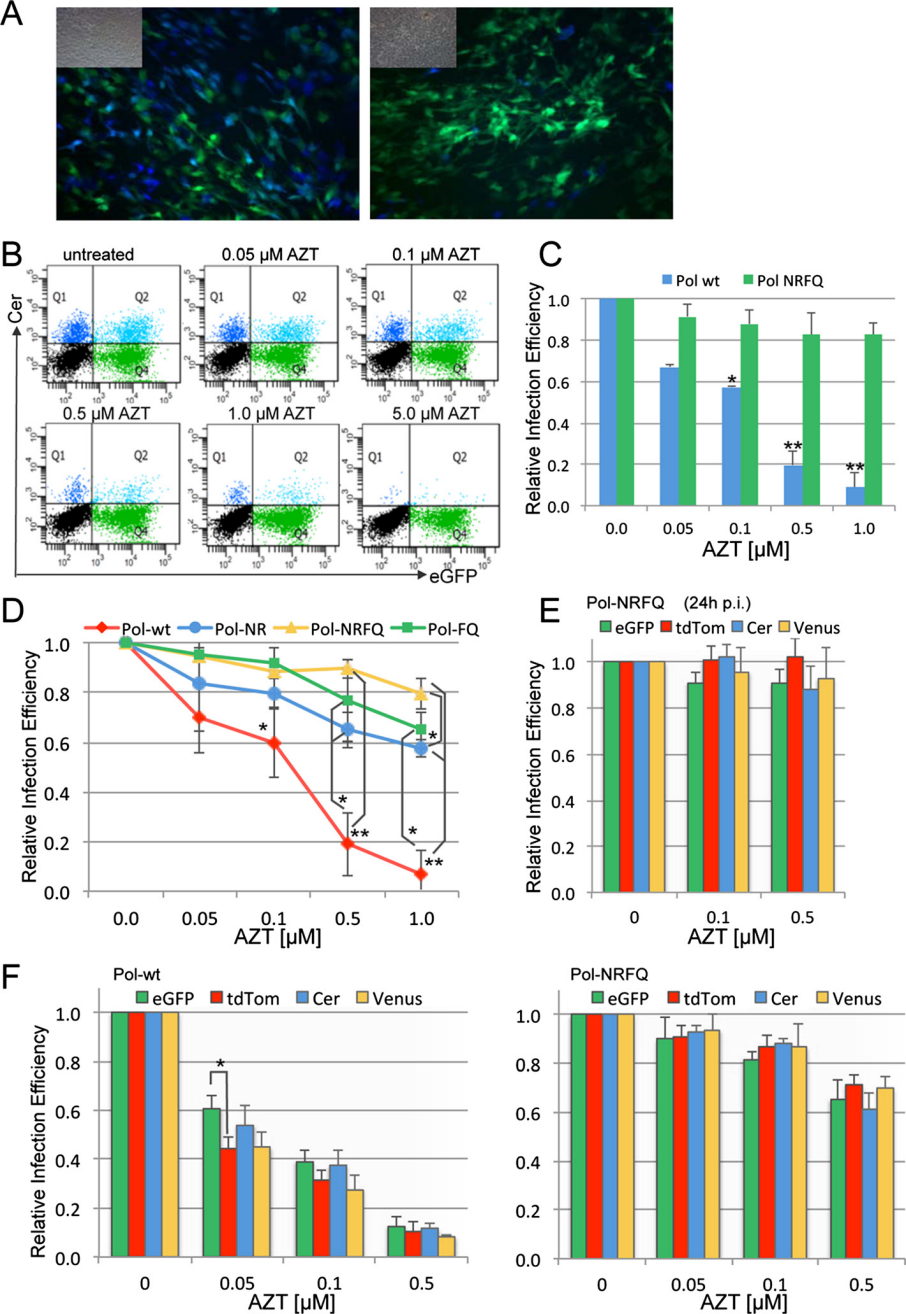
**The use of multi-colour LeGO vectors facilitates simultaneous analysis of different RT variants in single assays.** (**A**) Fluorescence microscopy demonstrates that vectors encoding Cerulean (packaged using a wild-type RT gene) or eGFP (packaged using the quadruple RT mutant [D67N/K70R/T215F/K219Q = NRFQ]) efficiently transduce SC1 cells (left panel), but only the vector packaged with the mutant RT facilitates efficient transduction in the presence of AZT (right panel). (**B**) AZT-sensitivity of the two RT variants in a dose-dependent manner was determined by FACS analysis. (**C**) Quantification of the FACS data from two independent experiments performed in duplicate. (**D**) Relative transduction efficiencies of four different lentiviral pseudotypes with the indicated Pol proteins (wild-type [wt], double mutants [D67N/K70R = NR, T215F/K219Q = FQ] or quadruple mutant (NRFQ) marked by tdTomato (red), Cerulean (blue), eGFP (green), or Venus (yellow), respectively. SC1 cells were transduced simultaneously with equivalent viral titres with or without AZT and analyzed 96 h post transduction. Transduction frequency ranged from 20 to 70 %. Shown are the mean values from two independent experiments. (**E**) AZT per se does not alter the detection sensitivity for any of the four fluorescent proteins tested. Human 293T cells were infected with lentiviral pseudotypes packaged with the quadruple RT mutant and containing a vector encoding the indicated fluorescent protein [eGFP, tdTomato (tdTom), Cerulean (Cer), or Venus]. AZT was added at the indicated concentration 24 h post transduction and analysed 96 h later. Shown are the mean values of four independent experiments. (**F**) The comparability of different fluorescent vectors was confirmed by assaying the sensitivity of Pol wt or the quadruple RT mutant (Pol NRFQ) to various concentrations of AZT with vectors encoding the indicated markers after transduction of human 293T cells and analysis 96 h post transduction. Transduction frequency ranged from 20 to 55 %. Shown are the mean values of a minimum of three experiments for each vector. P-values that were significantly different between all other data groups, or only for those connected by lines, are denoted with *, for values < 0.05 or **, for values < 0.01.

In the first experiment, particles harbouring wild-type RT transduced the blue fluorescent protein Cerulean, while particles harbouring the quadruple RT mutant (D67N/K70R/T215F/K219Q) transduced eGFP. As illustrated in Figure 2A (left panel), co-transduction of SC1 cells with both “wild-type” and “mutated” vectors resulted in a mixture of cells expressing eGFP and/or Cerulean. However, in the presence of 1 μM AZT only very few blue cells are visible, while the overall viability and absolute numbers of green cells was unchanged (Figure 2A, right panel). This observation was confirmed using FACS analysis (Figure 2B). As clearly evident, the parallel assay provides a sensitive and controlled assessment of drug sensitivities for different RT variants, which can be easily quantified (Figure 2C).

Finally, we co-transduced 293 T cells with four different LeGO vector particle types – containing wild-type RT or one of the three named mutants (two double mutants, one quadruple mutant), each marked with a different fluorescent tag (tdTomato, eGFP, Venus, or Cerulean) and pseudotyped with VSV-G protein. Transduction efficiencies were determined for each colour at various concentrations of AZT by FACS analysis. In this simple assay, differential drug sensitivity of four different *Pol* proteins could be assessed simultaneously – underlining the strength and convenience of the assay (Figure 2B). Furthermore, as viral transductions are performed on the identical cell population, variations in such assays that occur due to differences in cell growth are eliminated. A number of additional control experiments were performed to ascertain that drug addition (e.g. AZT) *per se* did not alter the fluorescent signal of the marker protein (Figure 2E) and that results obtained using different fluorescent markers were comparable (Figure 2F). In this latter assay, comparable results between the four fluorescent proteins were obtained for either the highly sensitive Pol wt or the relatively resistant quadruple mutant Pol when tested with different AZT concentrations (Figure 2F).

Taken together, this data demonstrates that the assay can be used to simultaneously test and/or screen (potential) antiviral compounds against multiple RT mutants using up to four fluorescent proteins. Moreover, the applicability of ecotropic Env proteins and thus the compatibility with Biosafety Level 1 facilities have been shown.

### Evaluation of the compatibility of the assay for large-scale drug screening

In the final step, we asked whether the method could readily be transferred to large-scale compound-screening systems. To do so, we evaluated the assay in a 384-well plate format applying an industrial high-throughput screening (HTS) read-out system. Test plates were automatically imaged and analysed using the PE Opera confocal LSM and the Acapella 2.0 software package. The z’ values of the plates were all above 0.3, which is well acceptable for cellular screens [11].

**Figure 3:**
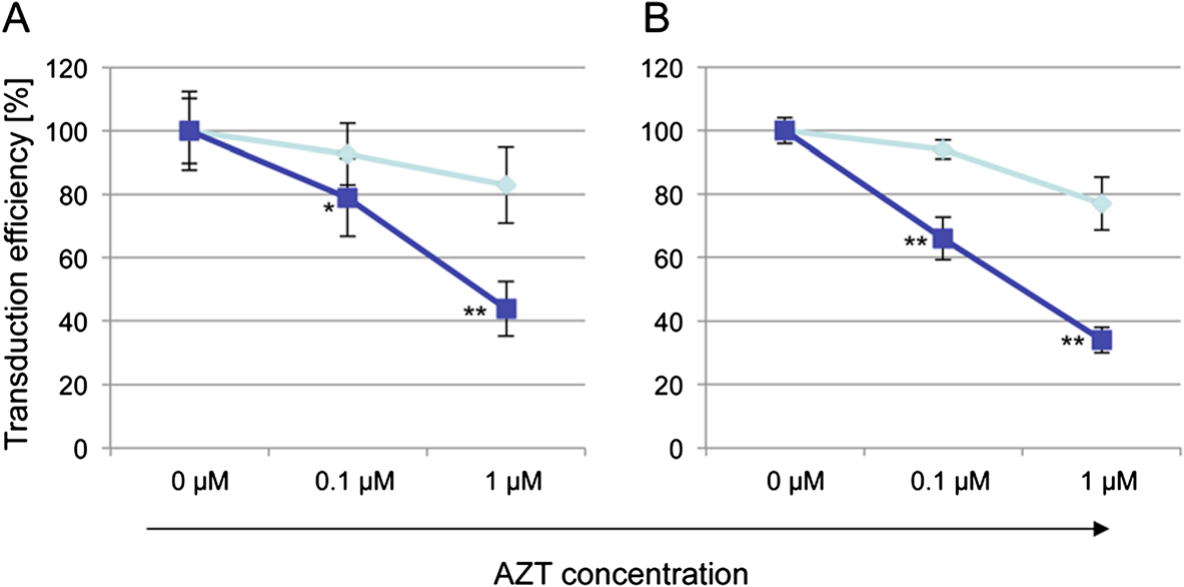
**Applicability for High-throughput screening (HTS).** Drug-sensitivity of wild-type (Pol wt) and mutant gag-pol (Pol NRFQ = D67N/K70R/T215F/K219Q) proteins was tested using HTS (**A**) as compared to conventional FACS screening (**B**). As exemplarily shown for AZT, very similar results were obtained with both methods. GFP expressing wild-type strain (filled squares/dark blue) and mCherry-expressing quadruple mutant strain (filled diamonds/light blue) are shown. HTS analysis was based on 2,400 cells whereas 10,000 cells were analysed by FACS. Note that both vectors were pseudotyped with ecotropic envelope protein compatible with S1 biosafety environment. P-values that were significantly different between the second data group are denoted with *, for values < 0.05 or **, for values < 0.0001.

We then compared the HTS assay set-up with results obtained from individual compound testing. As illustrated in Figure 3 for AZT, we found a very good correlation (99.91% at lowest). The HTS data show a slightly higher standard deviation due to the lower number of analysed cells, but the differences in 1 μM AZT susceptibility between wild-type and mutant RT pseudotypes were highly significant (p <0.001). Taken the reduction in cost, material and time consumption into account high-throughput screening will be a practical and efficient alternative to FACS.

## Conclusions

We provide here proof of principle for a novel technique that facilitates the testing of potential novel anti-HIV drugs simultaneously against different variants of the virus, as exemplary shown for *gag-pol* mutants. Since any compound with the potential to overcome a given escape mutation will be detected in our assay, it allows the identification of novel drugs targeting different key functions of HIV life cycle. Moreover, even molecules interfering with cellular factors restricting HIV infection would be identified in this assay. Thus drugs targeting all viral and cellular factors involved in virus entry, intracellular trafficking, reverse transcription, nuclear import, and integration can be screened. Furthermore, the protocol can be easily modified to test newly identified HIV mutants.

Adaptation of the technique to Biosafety Level 1 conditions has allowed successful upscaling to a high-throughput screening protocol under industrial conditions. In addition, inclusion of multiple virus variants (up to four, in the given study) into one assay will significantly reduce costs of large-scale screenings. Together, these two factors should make the proposed technique attractive for companies in both developed and developing countries. In conclusion, we suppose that the presented methods will become very valuable for the identification of novel anti-HIV drugs.

## Methods

All molecular cloning was performed using standard techniques. A DpnI-mediated site-directed mutagenesis [12] approach was used to introduce point mutations conferring AZT resistance into the *gag-pol* gene [9] in the packaging vector pMDLg/pPRE [10]. Standard cell culture methods were used as outlined previously [13]. Pseudotyped lentiviral LeGO vector particles were generated as described [7]. Standard-scale transduction procedures of different target cells followed the spinoculation protocol described earlier [7]. FACS analysis of one to four simultaneous infections was performed on FACS Aria using a 405 nm laser for detection of Cerulean (457/50 filter) and a 488 nm laser for detection of eGFP (510/20 filter), Venus (550/30 filter), and tdTomato (610/20 filter) (BD Biosciences). For large-scale screening in 384-well-plates, 3000 SC1 cells per well were incubated, without centrifugation and without polybrene, in the presence of virus for 48 h. Imaging was performed using a PE Opera confocal LSM after fixation with 3% formaldehyde and nuclei staining (Hoechst33342; 1.5 μM) at 20 fold magnification. Images were analysed on single cell level using Perkin Elmer Acapella 2.0. Single-cell data of each well were averaged and used for further analysis. Two-tailed, unpaired Student’s *t*-test was used to evaluate significance of data groups.

## Competing interests

B. Ellinger is an employee of the European Screening Port. This company is involved in the development and performance of large-scale drug screening assays. All other authors declare no conflict of interests.

## Authors’ contributions

MMP, KR, DVY, PVS performed small-scale experiments with lentiviral vectors and antiviral drugs; AD performed FACS analysis. KR and BE adapted the system to large-scale screening. BF, CS and VSP conceived and designed the experiments and drafted the manuscript. All authors read and approved the final manuscript.
